# Traumatic Proximal Rectus Femoris Avulsion With Recurrent Intramuscular Hematoma in a Competitive Martial Arts Athlete: A Case Report

**DOI:** 10.1155/cro/4860185

**Published:** 2026-10-05

**Authors:** Elise Bouchal, Kashani Mehdi, Kostas Economopoulos, David M. Melville, Douglas Rappaport, Aaron Thomas

**Affiliations:** ^1^ Alix School of Medicine Mayo Clinic Phoenix Arizona USA mayoclinic.org; ^2^ Department of Emergency Medicine Mayo Clinic Phoenix Arizona USA mayoclinic.org; ^3^ Department of Orthopedics Mayo Clinic Phoenix Arizona USA mayoclinic.org; ^4^ Department of Radiology Mayo Clinic Phoenix Arizona USA mayoclinic.org

**Keywords:** athletic trauma, bleeding disorders, case report, Factor VIII deficiency, intramuscular hematoma, quadriceps injury, rectus femoris avulsion, sports medicine imaging

## Abstract

Proximal avulsion of the rectus femoris tendon is an uncommon injury in athletes and is rarely associated with direct trauma and extensive hematoma. We describe a 25‐year‐old competitive taekwondo athlete who developed acute hip flexor weakness and thigh swelling after a direct anterior thigh kick. Bedside musculoskeletal ultrasound demonstrated disruption of rectus femoris architecture with intramuscular hematoma, and magnetic resonance imaging confirmed complete avulsion of both proximal heads of the rectus femoris with tendon retraction and a large associated hematoma. The patient had sustained a recent prior quadriceps injury with hematoma. Recurrent hematoma development warranted hematologic testing, which revealed an isolated Factor VIII deficiency. Given his high functional demands, he underwent open surgical repair. This case highlights the importance of early imaging and consideration of operative management in high‐level athletes presenting with trauma‐related rectus femoris avulsion. It also highlights the importance of hematologic workups in athletes presenting with unexplained recurrent hematomas to effectively risk‐stratify surgery and return to sport.

## 1. Introduction

Injuries involving the hip flexor muscles are common in professional sports, with estimates ranging from 5% to 28% of all injuries in high‐risk athletic populations. The primary muscles involved in hip flexion include the iliacus, psoas major, psoas minor, pectineus, rectus femoris (RF), and sartorius [1]. Among these, avulsion and rupture of the proximal RF are rare and account for only 0.5%–1.5% of hip injuries reported in athletes. Of those reported, the vast majority result from noncontact sports, and little is known about trauma‐induced avulsions [2, 3]. For a complete list of the RF avulsion injuries found in the literature, please see Table S1 [4–12,].

The RF tendon is especially at risk during kicking and running sports because it spans two joints, assisting in hip flexion and knee extension. The proximal attachment of this muscle consists of a direct head originating at the anterior inferior iliac spine (AIIS) and an indirect/reflected head originating at the anterior/superior acetabular rim and hip capsule. Distally, it joins the quadriceps tendon and inserts into the proximal pole of the patella to help extend the knee [1].

Athletes generally report sudden pain and a tearing sensation with an avulsion injury, which can involve one or both proximal heads. Immediately following the injury, athletes report either a normal gait or a slight limp [13, 14]. They generally present to the clinic with swelling and ecchymosis on the anterior thigh, weakened quadriceps extension against resistance, and discomfort with leg raising [2, 14, 15]. Risk factors for this type of injury to the RF include prior injury to the quadriceps and hamstring muscles and an athlete height of less than 182 cm [2].

Here, we describe a unique case of a 25‐year‐old, 172 cm, high‐performance taekwondo athlete with a previous left quadriceps injury who presented with complete avulsion of the left proximal RF, initially identified with point‐of‐care ultrasound. The injury involved both the direct and indirect heads, along with accompanying hematoma and edema of the proximal RF muscle.

This case contributes to the existing literature in several distinct and complementary ways. Proximal RF avulsions have been reported almost exclusively in soccer and American football athletes sustaining injury during self‐generated kicking or sprinting. In contrast, the present case appears to document the first complete dual‐head proximal RF avulsion resulting from a direct extrinsic blow sustained during competitive taekwondo—representing a fundamentally different injury mechanism [2]. The recurrent formation of massive intramuscular hematomas following combat sport trauma served as the clinical red flag that ultimately led to the diagnosis of mild hemophilia A, with a Factor VIII level of 25%–30%, a condition that frequently remains undetected until later in life when disproportionate surgical or posttraumatic bleeding prompts evaluation [16]. The initial hematoma episode, which had occurred earlier in the same year and was managed nonoperatively without evidence of tendon rupture, likely contributed to progressive weakening of the musculotendinous unit and predisposed the patient to the subsequent complete avulsion. This sequence illustrates a cumulative injury model in which an occult coagulopathy drives recurrent hematoma formation, progressive structural compromise, and ultimately catastrophic tendon failure, a pathophysiologic mechanism not previously described in the literature [2, 3, 13]. The disproportionate postoperative hematoma formation and persistent wound drainage following suture anchor repair further underscored the presence of an underlying bleeding diathesis, reinforcing the importance of maintaining a high index of suspicion for coagulopathy when postoperative bleeding exceeds clinical expectations following musculoskeletal surgery [16]. This case also highlights an important gap in the current literature regarding return‐to‐sport guidance for elite combat athletes with newly diagnosed hemophilia A. While existing recommendations address prophylaxis for sport participation in general terms, no framework exists to guide return to full‐contact martial arts—a discipline characterized by repeated high‐energy direct muscle trauma, necessitating individualized prophylactic Factor VIII strategies and avoidance of antiplatelet agents.

## 2. Case Presentation

### 2.1. Patient Care Timeline

• September 2025: progressive left thigh pain/swelling after sparring; MRI 9/18 demonstrated hematoma; conservative management.
• November 2025: severe martial arts injury; 11/20 imaging demonstrated complete RF avulsion with hematoma involving the vastus medialis; surgical repair 11/28 with evacuation of ~1 L hematoma.
• December 2025: persistent postoperative bleeding/recurrent hematoma; 12/17 recurrent pain/tightness led to incision and drainage; postoperative Hb nadir 10.4 g/dL without transfusion.
• January 2026: hematology evaluation; ISTH‐BAT 6; FVIII 25%, repeat 30%; inhibitor negative; VWF evaluation normal.

### 2.2. Patient Presentation and Care Details

A 25‐year‐old male competitive taekwondo athlete presented with acute left anterior thigh and hip pain following direct trauma sustained during a competitive event. During sparring, he was kicked forcefully in the anterior aspect of his left thigh, his lead leg, near the proximal quadriceps region. He experienced immediate severe pain accompanied by rapid and marked swelling of the thigh and a consequent loss of hip flexor function, which significantly impaired his ability to ambulate.

During evaluation in the sports medicine clinic, physical examination revealed a markedly enlarged left thigh with visible swelling and ecchymosis extending into the proximal thigh and groin. Palpation demonstrated significant tenderness and fluctuance over the anterior proximal thigh. Hip flexion was severely limited due to pain and weakness, whereas knee extension was notably weakened. Distal sensation, motor function, and peripheral pulses were intact.

Point‐of‐care musculoskeletal ultrasound of the left anterior thigh was subsequently performed and revealed disruption of the normal RF muscle architecture with surrounding edema and a hypoechoic focal fluid collection in the region of the proximal muscle belly (Figure 1). Color Doppler evaluation showed no internal vascularity suspicious for mass. These findings raised concern for significant musculotendinous injury and prompted further evaluation with magnetic resonance imaging.

**Figure 1:**
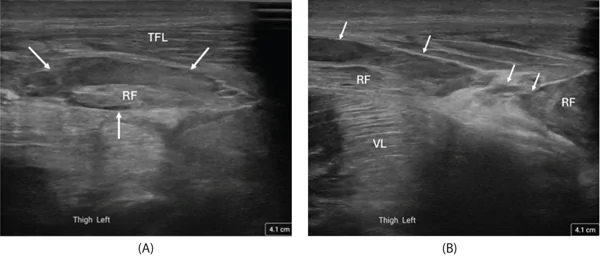
Ultrasound of anterior thigh. Long axis images at the (A) proximal and (B) distal aspects of the hypoechoic collection (arrows) show focal fluid tracking around disrupted muscular architecture of the rectus femoris, situated between the tensor fascia lata (TFL) and vastus lateralis (VL).

Magnetic resonance imaging of the left femur and pelvis was subsequently obtained and demonstrated a complete avulsion of the RF tendon at its proximal origin involving both the direct and indirect heads, with approximately 5 cm of proximal tendon retraction (Figure 2)A. A large associated perimuscular and anterior proximal thigh hematoma was identified, extending circumferentially around the proximal RF (5.1 × 5.7 cm in maximum axial dimensions) and measuring up to 13.4 cm in craniocaudal dimension (Figure 2)B. Additional findings included a resolving intramuscular hematoma within the vastus intermedius muscle (Figure 3)A—with surrounding edema and early heterotopic ossification corresponding to earlier radiographic findings (Figure 3)B—as well as diffuse edema involving adjacent musculature, including the iliopsoas, sartorius, tensor fascia lata (TFL), and gluteal muscles.

**Figure 2:**
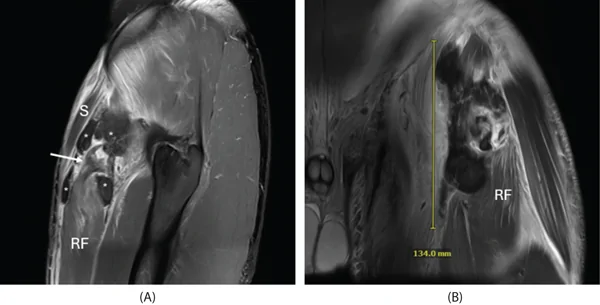
MR imaging of rectus femoris avulsion. (A) Sagittal PD fat‐saturated image shows the distally retracted rectus femoris (RF) tendon stump (arrow), avulsed from its pelvic origin, surrounded by low‐signal hemorrhage (∗) deep to the sartorius, with associated edema in the RF muscle. (B) Coronal T2 sat‐saturated image demonstrates the craniocaudal extent of the hemorrhage (calipers) and edematous, retracted muscle belly (RF).

**Figure 3:**
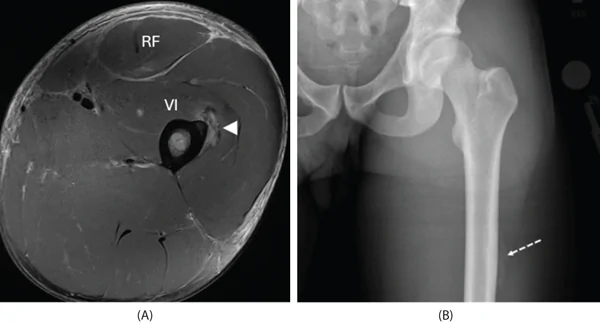
MR imaging of resolving muscle hematoma. (A) Axial T2 fat‐saturated image at mid‐thigh shows heterogeneous signal (arrowhead) within the lateral vastus intermedius (VI), representing residual blood products and early mineralization adjacent to focal cortical thickening. The adjacent rectus femoris (RF) muscle is uninjured. (B) Same‐day femur radiograph reveals heterotopic ossification (dashed arrow) corresponding to the MRI findings.

The patient′s clinical history was notable for a prior episode earlier in the same year involving the left thigh, during which he sustained a large traumatic hematoma (4.1 × 2.6 cm circumferential and measuring 8.0 cm in craniocaudal length) following a similar athletic activity. That injury was managed nonoperatively with observation, and earlier imaging from the prior event demonstrated hematoma formation without tendon rupture (Figure 4). The recurrent nature of these significant hematomas following relatively limited trauma raised concern for possible cumulative weakening of the musculotendinous unit or an underlying predisposition to bleeding.

**Figure 4:**
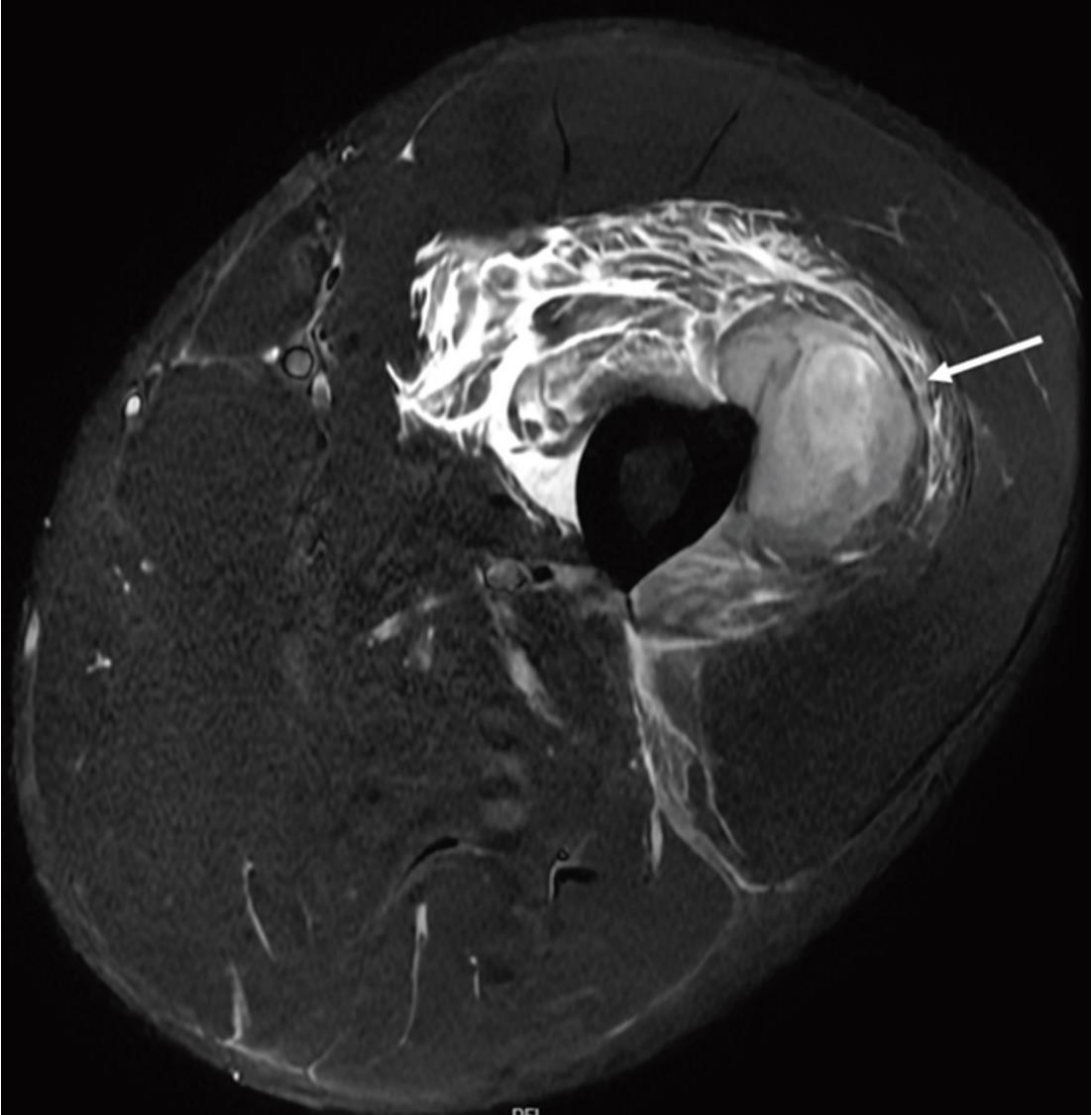
Prior vastus intermedius injury. Coronal STIR image shows pronounced vastus intermedius muscle edema with a hematoma (arrow) in the lateral muscle belly overlying focal cortical thickening.

Given the patient′s young age, high functional demands, and desire to return to elite‐level martial arts competition, operative management was recommended. After a detailed discussion of risks, benefits, and alternatives, the patient elected to proceed with surgical repair. After general anesthesia was induced, the left hip and thigh were prepped and draped in typical fashion. The anterior superior iliac spine (ASIS) was identified and marked. A 6‐cm longitudinal incision starting 2 cm distal and 2 cm lateral to the ASIS was carried distally, centered over the rectus tendon. Dissection through the subcutaneous tissue was performed until the underlying muscle fascia overlying the TFL and the sartorius was identified, paying special attention to protecting the branches of the lateral femoral cutaneous nerve (LFCN). The fascia overlying the interval between the TFL and sartorius was opened sharply, and the two muscle bellies separated, allowing visualization of the AIIS. A large hematoma was present at this level. Roughly 1 L of hematoma was evacuated from the area. Upon distal palpation, the proximal end of the torn direct head of the RF was identified and mobilized into the incision. The RF tendon edges were debrided to healthy tissue, and a #2 nonabsorbable suture was run up and down the lateral tendon in a Krackow manner. A second #2 nonabsorbable suture was run in a Krackow manner up and down the medial aspect of the tendon. All scar tissue was cleared off the AIIS. Both #2 nonabsorbable sutures were fixed to the AIIS using two bioabsorbable 4.75‐mm suture anchors, bringing the torn tendon in direct apposition with the AIIS. This was done with the hip in full extension, and a tension‐free repair was confirmed on palpation. After confirming the adequacy of the repair, the wound was irrigated with 1 L of normal saline. Hemostasis was obtained with electrocautery. The incision was closed in layers using 2‐0 Vicryl and 3‐0 Monocryl. A sterile dressing was applied. He was placed on crutches and instructed to remain 50% weight‐bearing for 6 weeks.

In the early postoperative period, the patient developed recurrent wound drainage consisting of dark sanguineous fluid, consistent with postoperative hematoma formation. He denied systemic symptoms such as fever or chills. Examination revealed a small area of incisional dehiscence with ongoing hematoma drainage, but no signs of purulence or deep infection. Conservative management with compression dressings and close follow‐up was initiated, and empiric oral antibiotics were prescribed for prophylaxis. The patient reported gradual improvement in pain and swelling with this approach.

Given his history of recurrent large hematomas following minor trauma and postoperative bleeding disproportionate to the expected surgical course, a hematology evaluation was requested. As outlined in the attached hematology communication, a comprehensive coagulopathy workup was recommended prior to formal consultation—including complete blood count with differential, prothrombin time, activated partial thromboplastin time, and fibrinogen level—to evaluate for a possible underlying bleeding diathesis.

A CBC demonstrated a normal platelet count (283 × 10^9^/L) and a hemoglobin of 12.6 g/dL. Coagulation studies showed a PT of 10.8 s, INR of 1.0, aPTT of 35 s, thrombin time of 22.7 s, and fibrinogen level of 230 mg/dL. Factor VIII activity was reduced to 25% and remained low at 30% on repeat testing, whereas Factor IX activity (101%) and Factor XIII testing were normal. Von Willebrand factor (VWF) antigen (73%), VWF activity (64%), and VWF binding were normal, and testing for a Factor VIII inhibitor was negative. These findings were most consistent with mild congenital hemophilia A and argued against acquired hemophilia and type 2N von Willebrand disease.

His bleeding history was notable for recurrent traumatic muscle hematomas and excessive bleeding following tooth extractions, with an ISTH bleeding assessment tool score of 6. He had no known family history of bleeding or clotting disorders and no history of spontaneous mucocutaneous bleeding, petechiae, or spontaneous ecchymoses. Medication review included acetaminophen and intermittent postoperative medications; aspirin was held, and avoidance of aspirin, NSAIDs, and other platelet‐inhibiting medications was recommended. The physical examination was otherwise unremarkable without evidence of systemic disease or active spontaneous bleeding.

Following diagnosis, hematology recommended Factor VIII pharmacokinetic testing and prophylactic Factor VIII replacement before high‐risk martial arts activity. At the latest follow‐up, the patient remained neurovascularly intact with improving pain control and preserved range of motion of the hip and knee. He was counseled on activity modification and the anticipated course of postoperative rehabilitation, with plans for structured physical therapy. The incidental diagnosis of mild hemophilia A has greatly impacted his recovery and return to sport. He was strongly advised to administer prophylactic Factor VIII prior to martial arts contact sessions, and to avoid high‐impact contact until this prophylaxis strategy has been determined. Treatment options discussed included Adynovate, Esperoct, and Eloctate. He was also advised to avoid NSAIDs, aspirin, and platelet‐inhibitors to decrease bleeding risk. The patient is currently working with a hematologist to determine treatment for the bleeding disorder and ability to return to sport. The patient has participated in physical therapy and is able to bear weight on the leg; however, further return to sport has been delayed due to the diagnosis of mild hemophilia A.

## 3. Discussion

Due to rarity and limited data, there is some controversy as to whether surgery should be considered for patients who present with avulsion of the proximal heads of the RF [2, 15, 17]. In both nonoperative and surgical approaches, outcomes of RF avulsions are generally good, with a mean return to play of 69.2 days and 3.8 ± 0.8 months, respectively. However, studies focused on nonoperative approaches have reported that athletes were not able to return to complete baseline function postinjury, which may be due to calcification of the avulsed tendon stump. Conversely, a study that followed four patients all of whom did undergo surgery noted a full return to preinjury level of function at 15.8 ± 2.6 weeks [18]. In a review of 132 athletes with full‐thickness RF avulsion cases, those who underwent operative repair had a 100% return to play rate versus 93.3% in those who chose to pursue nonoperative treatments [19]. In our case, the patient was concerned with his ability to return to both sport and baseline function and therefore pursued surgical treatment. Treatment postsurgery in the literature involves placement of a knee brace that is locked in extension for 2–4 weeks, physical therapy, and return to sport generally between 4 and 6 months following the operation [20].

Our case was further complicated by known risk factors, including a spontaneous hematoma injury developed after a sparring session in sport 2 months prior to avulsion, along with a shorter stature. The resolving hematoma shown on his MRI was located within the vastus intermedius muscle and did show signs of healing at the time of the evaluation for the avulsion injury. A study that explored Australian football injuries found that a previous quadriceps injury correlated with an increased risk of injury to the RF, possibly due to the change in gait and weakness of the muscle following the injury. This study also noted that players below 182 cm were at a higher risk of injury to the quadriceps muscle [21, 22]. Our patient, with a height of 172 cm, demonstrated both risk factors.

A traumatic mechanism involving direct impact is rare in reported cases and is likely related to the nature of the patient′s sport. Traumatic injuries often involve the formation of hematoma, and this was reported in his prior quadriceps injury and the current avulsion injury. It is likely that a direct impact of an opponent′s kick to the front leg that was flexed at the hip to about 90° caused this hematoma, along with the subsequent loss of function in walking and leg raise [23]. In reviews of full‐thickness avulsions, there is limited discussion of the nature of injury, and most describe kicking (47.6%; 30/63), excessive knee flexion and forced hip extension (42.9%; 27/63), and sprinting (9.5%; 6/63) [18]. Due to the lack of cases reporting specific trauma‐involving avulsion injuries, little is known as to how the hematoma might have affected the healing of the injury.

Due to the limited number of cases in RF avulsions, little is known about hematoma reformation following surgery. However, postoperative complications include hematomas that are not commonly seen in other hip surgeries. In a review of primary total hip arthroplasty, it was reported that of the 149,026 patients who underwent primary THA, 180 (0.12%) required a second surgery due to postoperative hematoma, and that these patients were significantly at higher risk of deep wound infection [24]. Our patient did not classify under the risk factors of high BMI > 35 or prior known history of bleeding disorder that were listed in the review; however, it may be possible that he was at higher risk of infection due to the postoperative hematoma. This further highlights the importance of testing patients for hematologic abnormalities when recurrent hematomas are noted in injury progression.

Aside from the rarity of a trauma‐induced injury, this case also denotes the importance of testing for bleeding disorders in athletes with recurrent hematomas. Due to the high‐impact nature of taekwondo, athletes with bleeding disorders are at high risk of dangerous bleeding complications and have generally been discouraged from engaging in high‐impact sports [16]. The loss of efficient clotting and/or platelet function increases the risk for dangerous bleeding in response to trauma and injury in these patients. In mild hemophilia A, patients are at an increased risk of bleeding and chronic hemarthrosis due to a deficiency (< 40%) in clotting Factor VIII. This diagnosis increases the risk of bleeding episodes and related complications, further increases the complexity of surgical treatments, and has historically limited involvement in high‐risk contact sports [16]. This case describes the clinical decision to order hematologic testing following recurrent hematomas, which revealed a Factor VIII deficiency and mild hemophilia A diagnosis.

Athletes with known bleeding disorders, especially mild hemophilia A due to persistent FVIII deficiency, have historically been discouraged from engaging in high‐impact sports due to risk of bleeding and complex injuries. Furthermore, the complexity of surgery is further increased in patients with bleeding disorders and may contribute to a patient′s decision to pursue surgical repair [16]. Due to the advising against contact sports in patients with hemophilia A, there is very little known about athletes with this disorder competing in sports, and no reports about athletes competing in taekwondo. There have been recent case reports that note success in athletes with this bleeding disorder competing in soccer and basketball at the high school and collegiate level; however, these athletes relied heavily on prophylactic FVIII replacement therapies [19, 25]. The necessity of developing a prophylaxis protocol severely limits return to sport as was noted in this case report and is imperative to protect the athlete in and out of the sport.

To conclude, this is a rare occurrence of a trauma‐induced avulsion of the direct and indirect proximal heads of the RF in a high‐performing athlete with a history of quadriceps injury. The patient reported hip flexor pain, difficulty walking, development of a limp, and numbness and tingling in his toes while lying down. He pursued an open repair of his left rectus tendon due to his desire to return to a highly kicking sport. His case was further complicated by a previous resolving hematoma in the adjacent left vastus intermedius muscle at the time of injury and an accompanying hematoma and edema in his RF following the avulsion injury. Following these hematomas, this athlete was tested for and diagnosed with mild hemophilia A. This case highlights the importance of testing athletes presenting with recurrent hematomas for bleeding disorders to better inform surgical repair options, return to sport protocol, and general patient safety.

## 4. Conclusion

This case highlights a rare, trauma‐induced avulsion of both the direct and reflected proximal heads of the RF in an elite martial arts athlete. The injury occurred in the setting of direct impact and was associated with extensive intramuscular hematoma and surrounding soft tissue edema, likely influenced by a recent prior quadriceps injury. Given the patient′s high functional demands and desire to return to competitive sport, operative repair was pursued.

This case underscores the importance of maintaining a high index of suspicion for proximal RF avulsion in athletes presenting with acute hip flexor weakness and thigh swelling after direct trauma. Early use of bedside ultrasound followed by advanced imaging can facilitate timely diagnosis. Additionally, the presence of recurrent or disproportionate hematoma warrants further investigation with a hematological workup to investigate any potential bleeding disorders, and is imperative for informing treatment options and patient safety. Future studies are needed to better define the role of traumatic hematoma and prior muscle injury in the pathophysiology, management, and outcomes of proximal RF avulsions.

## Funding

No funding was received for this manuscript.

## Consent

No written consent has been obtained from the patients, as no patient‐identifiable data are included in this case report.

## Conflicts of Interest

The authors declare no conflicts of interest.

## Supporting information


**Supporting Information** Additional supporting information can be found online in the Supporting Information section. For a complete list of the rectus femoris avulsions found in the literature, please see Table S1. Table S1: Literature review concerning past descriptions of rectus femoris avulsion injury.

## Data Availability

The data that support the findings of this study are available on request from the corresponding author. The data are not publicly available due to privacy or ethical restrictions.
