# Supplementary material for: Traumatic Proximal Rectus Femoris Avulsion With Recurrent Intramuscular Hematoma in a Competitive Martial Arts Athlete: A Case Report
Source: Case Rep Orthop. 2026 Oct 5;2026:4860185. doi: 10.1155/cro/4860185 (PMC13639391; doi:10.1155/cro/4860185)
Supplement: Supplementary file 1 [file CRO-2026-4860185-s001.docx]

Table 1: Literature review concerning past descriptions of rectus femoris avulsion injury

| **Study** | **Study Design** | **N** | **Age / Sex** | **Sport** | **Mechanism** | **Injury Location** | **Treatment** | **Return to Sport (RTS)** | **Key Outcomes** | **Reference** |
| --- | --- | --- | --- | --- | --- | --- | --- | --- | --- | --- |
| **Irmola et al. (2007)** | Case series | 5 | Median 21 y (19–27); all male | Soccer (4), hurdler (1) | Eccentric contraction (kicking) | Proximal tendon (complete avulsion from AIIS) | Surgical suture anchor repair (2–4 anchors); surgery delayed 18–102 days | 5–10 months | All returned to pre-injury level; all rated "good"; 1 patient developed ectopic calcification | **18** |
| **Gamradt et al. (2009)** | Case series (NFL Injury Surveillance) | 11 | Professional NFL athletes | American football | Kicking and sprinting | Proximal rectus femoris | Nonoperative | Mean 69.2 days (~6–12 weeks) | All returned to unrestricted play; incidence ~1 case/year after MRI adoption | **8** |
| **Esser et al. (2015)** | Case report | 1 | 24 y; male | NCAA Division I soccer (goalkeeper) | Goal kick | Proximal complete avulsion | Nonoperative rehabilitation following ultrasound diagnosis | Full recovery; no recurrence | Demonstrated ultrasound as an effective diagnostic tool; supports nonoperative management in elite kicking athletes | **5** |
| **Ueblacker et al. (2015)** | Case series | 4 | Mean 30 ± 2 y; all male | Professional European soccer | Kicking (dominant leg) | Proximal complete avulsion with significant retraction | Suture anchor repair; surgery at mean 60 ± 88 days | RTP: 111 ± 15 days; RTC: 140 ± 23 days | All returned to same competitive level; no recurrence at 35-month follow-up; MRI confirmed anatomic reinsertion | **19** |
| **Baumgart et al. (2021)** | Case report | 1 | 41 y; male | Recreational endurance athlete | Eccentric mechanism | Proximal complete avulsion | Surgical repair | Endurance performance recovered by 6 months | Persistent neuromuscular asymmetries and compensations at 1 year despite functional recovery | **20** |
| **Kayani et al. (2021)** | Cohort study | 55 (25 repair, 30 tenodesis) | Professional athletes; 48 male, 7 female | Various professional sports | Kicking/sprinting | Proximal rectus femoris | Surgical repair vs. primary tenodesis | Repair: 15.8 ± 2.2 wk; Tenodesis: 12.4 ± 1.6 wk | All returned to preinjury level; tenodesis had faster RTS, 0% recurrence vs. 16% after repair; similar strength and satisfaction at 2 years | **21** |
| **Salazar & Kollmorgen (2021)** | Case report + systematic review | 1 case + 64 reviewed | Not specified | Various sports | Various | Proximal rectus femoris | Endoscopic repair; systematic review included nonoperative, suture repair, anchor repair, and excision | Good outcome (endoscopic case) | First reported endoscopic repair; literature complications included lateral femoral cutaneous nerve injury and wound complications | **22** |
| **Hinz et al. (2022)** | Retrospective cohort | 16 (of 20 enrolled) | All male; 87.5% soccer | Soccer | Kicking | Acute proximal rectus femoris avulsion (<6 weeks) | Suture anchor refixation; surgery at mean 18.4 ± 8.5 days | Mean 10.5 ± 3.4 months | 93.3% returned to competition; no strength deficit; TAS 9; HHS 100; 93.8% satisfied; no complications | **23** |
| **McAleer et al. (2022)** | Retrospective cohort (9 years) | 38 injuries in 27 athletes (4 surgical grade 4c) | Mean 24.7 ± 2.3 y; 10 male, 17 female | Elite track & field | Sprinting (surgical cases) | Proximal free tendon (grade 4c) | Rehabilitation for most; surgery for complete grade 4c injuries | Rehab: 20.4 days; Surgery: 129.8 ± 16.8 days | All surgical cases were female sprinters; no recurrence after surgery; grade 3 injuries had higher reinjury rates | **24** |
| **Holder et al. (2021)** | Case report | 1 | Not specified | Non-sport (trauma) | Trauma | Distal rectus femoris myotendinous junction | Operative repair | Not specified | Extremely rare distal injury; emphasizes diagnostic challenges and lack of standardized treatment | **25** |
